# Serum metabolic signatures associated with running performance in healthy men living in a cold environment: insights into amino acid and energy metabolism

**DOI:** 10.3389/fphys.2026.1920016

**Published:** 2026-10-01

**Authors:** Kexin Fang, Lixia Yu, Ruixin Zhu, Jingjing He, Liwei Zhang, Ran Wang, Yiran Zheng, Tieqiang Sun, Zhanxin Yao, Jie Guo

**Affiliations:** 1 Key Laboratory of Precision Nutrition and Food Quality, Department of Nutrition and Health, China Agricultural University, Beijing, China; 2 Military Medical Sciences Academy, Academy of Military Sciences, Tianjin, China; 3 Peking University Third Hospital, Beijing, China; 4 Department of Clinical Neuroscience, Karolinska Institutet, Stockholm, Sweden

**Keywords:** exercise physiology, running performance, metabolomics, glutamic acid, cortisol, amino acid metabolism, energy metabolism

## Abstract

**Background:**

Chronic exposure to cold environments may impose distinct metabolic demands that are relevant to physical performance; however, the metabolic characteristics associated with physical performance in individuals living in cold environments remain incompletely understood. We aimed to characterize serum metabolomic profiles associated with differences in physical performance among healthy adults residing in a cold region.

**Methods:**

Among 178 healthy males residing in a cold-region environment, 141 participants with available serum metabolomics data were included and stratified according to 3,000-meter running performance into Good (≤14 minutes 13 seconds, n = 76) and Poor (>14 minutes 13 seconds, n = 65) groups. Fasting serum samples underwent untargeted ultra-performance liquid chromatography–tandem mass spectrometry profiling. Differentially abundant metabolites were identified using covariate-adjusted linear models implemented in the limma framework, with appendicular skeletal muscle mass index included as a continuous covariate. Differentially abundant metabolites were defined using a Benjamini–Hochberg FDR-adjusted *P* < 0.05 and |log_2_FC| > 0.25. Global metabolic profiles were explored using PCA and PLS-DA, followed by KEGG pathway enrichment analysis.

**Results:**

A total of 141 males aged 28–40 years were included. After adjustment for ASMI, 10 differentially abundant metabolites were identified between the Good and Poor groups. Six metabolites showed higher adjusted abundances in the Good group, including cortisol (FDR = 0.031, log_2_FC = 0.321, 95% CI: 0.155 to 0.488), whereas four metabolites showed higher adjusted abundances in the Poor group, including glutamic acid (FDR = 0.013, log_2_FC = −0.262, 95% CI: −0.380 to −0.145). Nominally significant enrichment was observed for nitrogen metabolism (*P* value = 0.011), arginine biosynthesis (*P* value = 0.026), butanoate metabolism (*P* value = 0.028), and histidine metabolism (*P* value = 0.030); however, no pathway remained significant after FDR correction.

**Conclusions:**

Differences in running performance were associated with distinct serum metabolic patterns involving amino-acid and nitrogen metabolism, phospholipid metabolism, and stress- and redox-associated metabolic processes. These findings may provide metabolic hypotheses on inter-individual differences in running performance and identify candidate metabolic pathways for further investigation.

## 1 Introduction

Physical performance is a functional indicator of health status and well-being, and encompasses multiple domains, including cardiorespiratory fitness, muscular strength, and physical capability (Cooper et al., 2010). Higher levels of physical performance have been associated with lower risks of cardiovascular disease and mortality (García-Hermoso et al., 2018; Veronese et al., 2018; Laukkanen et al., 2022). Understanding the biological basis of physical performance may provide insights into the metabolic processes associated with exercise capacity and may inform future targeted interventions.

Metabolomics offers a comprehensive approach for characterizing the small-molecule signatures that may underlie differences in physical performance (Schranner et al., 2020). Previous studies have identified circulating metabolites associated with multiple dimensions of physical performance, including gait speed, grip strength, cardiorespiratory fitness, and walking ability (Marron et al., 2020; Nierenberg et al., 2020; Kistner et al., 2021). These associations have involved lipid, amino-acid, and other energy-related metabolic pathways, suggesting that circulating metabolic profiles may reflect biological processes underlying inter-individual differences in physical performance.

Circulating metabolic profiles are also influenced by environmental conditions. Environmental temperature can alter thermoregulatory demands, energy expenditure, and substrate metabolism. Acute cold exposure has been associated with increased energy expenditure and brown adipose tissue activity in humans (Huo et al., 2022). Metabolomic studies have further demonstrated that acute cold exposure could alter circulating metabolic pathways, particularly those involved in amino-acid metabolism, pyruvate metabolism, and the tricarboxylic acid cycle (Kovaničová et al., 2021). These findings suggest that environmental temperature may contribute to variation in systemic metabolic profiles. However, metabolic profiles associated with physical performance among healthy adults living in cold environments remain poorly understood.

Therefore, this study investigated serum metabolomic profiles in healthy adults with divergent 3,000-meter running performance residing in a cold region. To account for potential confounding by muscle mass, metabolite differences were evaluated after adjustment for appendicular skeletal muscle mass index (ASMI). We aimed to characterize metabolic patterns and identify candidate metabolites and metabolic pathways associated with running performance.

## 2 Methods

### 2.1 Study population

This study was conducted on a population of 178 healthy male adults residing in a cold environment, where the coldest month has an average temperature of −10 °C to 0 °C, and 90~145 days in a year with an average daily temperature of ≤ 5 °C. All participants provided written informed consent, and the study protocol was approved by the Ethics Review Committee of the Military Medical Sciences Academy (No.: AF/SC-08/02.307), in accordance with the Declaration of Helsinki. After excluding participants without complete ASMI or metabolomic data, 141 participants were included for serum metabolomics profiling (**Figure 1**). The population was divided into two groups according to the median 3,000-meter running time: Good (≤ 14 min 13 s) and Poor (>14 min 13 s). For all participants, demographic characteristics were collected via questionnaires and body composition was assessed using the InBody270 bioelectrical impedance analysis (BIA) system.

**Figure 1:**
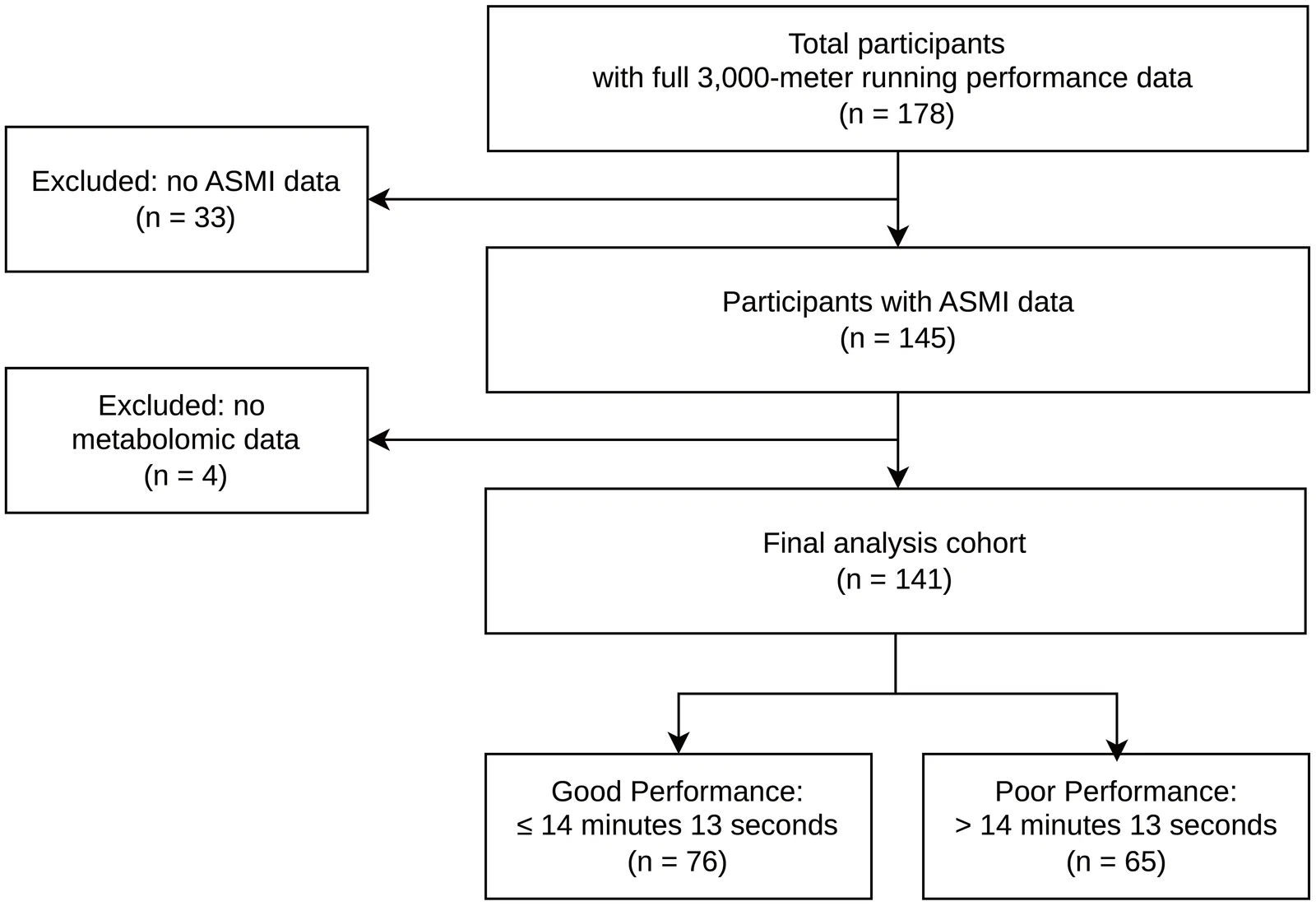
Study flow diagram. A total of 178 participants were initially recruited. After excluding those without complete appendicular skeletal muscle mass index (ASMI) and metabolomic data (n = 37), 141 participants were included in the final analysis. The Good and Poor groups were defined based on 3,000-m running performance.

### 2.2 Metabolomic profiling

Untargeted serum metabolomic profiling was performed according to established methods (Wang et al., 2022) and GB/T 44827-2024.

### 2.3 Sample collection and preparation

Fasting venous blood samples were collected from all participants between 7:00 and 9:00 AM following a minimum 12-hour fast. After clotting at room temperature (22 °C) for 30 minutes, serum was separated by centrifugation at 3,000 × g at 4 °C for 10 minutes. Aliquots of 100 μL serum were snap-frozen in liquid nitrogen and stored at −80 °C within 2 hours of collection; samples were never subjected to freeze–thaw cycles prior to analysis. After thawing on ice, 100 μL of each serum sample was mixed with 400 μL pre-cooled methanol in a 1.5 mL microcentrifuge tube, vortexed, and centrifuged at 13,000 × g at 4 °C for 15 minutes. The resulting supernatant was transferred for untargeted ultra-performance liquid chromatography-tandem mass spectrometry (UPLC–MS/MS) analysis.

### 2.4 Chromatographic and mass spectrometric analysis

Untargeted metabolomic profiling was performed using an UltiMate 3000 UPLC system (Thermo Fisher Scientific) coupled to a Q-Exactive high-resolution mass spectrometer. Chromatographic separation was achieved on an ACQUITY UPLC HSS T3 column (100 mm × 2.1 mm, 1.8 μm; Waters) maintained at 40 °C. The mobile phase consisted of solvent A (5 mM ammonium acetate and 5 mM acetic acid in water) and solvent B (acetonitrile), delivered at 0.3 mL/min. A 10-minute gradient was applied: 2% B (0–0.8 min), ramping to 70% B (0.8–2.8 min), 90% B (2.8–5.6 min), 100% B (5.6–8.0 min), returning to 2% B at 8.1 min with re-equilibration until 10.0 min. Mass spectra were acquired in both positive and negative ionization modes under data-dependent acquisition (DDA). Full-scan MS spectra (70–1050 m/z) were collected at a resolution of 70,000 (AGC target 3×10^6^, maximum IT 100 ms), with the top three precursors selected for MS/MS fragmentation at a resolution of 17,500 (AGC target 1×10^5^, maximum IT 80 ms). Pooled quality control (QC) samples were injected every 10 experimental samples to monitor instrument stability and analytical reproducibility. The relative standard deviation (RSD) of metabolic features across QC injections was calculated to assess analytical precision. The results of the QC assessment are presented in **Supplementary Figure 2**.

### 2.5 Data processing and metabolite annotation

Metabolite annotation was performed by matching detected features against the Human Metabolome Database (HMDB) and Kyoto Encyclopedia of Genes and Genomes (KEGG) databases with a mass tolerance of <10 ppm. Putative identifications were further evaluated based on isotopic distribution patterns and cross-referenced against an in-house fragment spectrum library where available. Drug-related metabolites, xenobiotics, and features with >20% missing values were excluded. Following feature filtering and metabolite annotation, 636 endogenous metabolites were retained for downstream statistical analysis.

### 2.6 Statistical analysis

Baseline characteristics were compared between the Good and Poor running-performance groups using the independent-samples t-test for continuous variables with approximately normal distributions, the Wilcoxon rank-sum test for non-normally distributed continuous variables, and Fisher’s exact test for categorical variables.

For metabolite-level statistical analysis, linear models were fitted separately for each metabolite using the limma framework in R, with ASMI included as a continuous covariate. Several participant characteristics have been reported to be associated with physical performance and circulating metabolite profiles, including age, body composition, physical activity, smoking, and other lifestyle and clinical factors (Zeiher et al., 2019; Bar et al., 2020; Kistner et al., 2021).ASMI was calculated as appendicular skeletal muscle mass (ASM) divided by height squared (kg/m^2^), consistent with the commonly used definition of skeletal muscle mass index (Chen et al., 2020). Skeletal muscle mass is closely related to physical performance and has been reported to be a strong predictor of cardiorespiratory fitness (Ren et al., 2023). Given the sample size, we selected ASMI as the primary covariate to maintain a parsimonious model. Sensitivity analysiswere performed using alternative covariate specifications, including age alone, physical activity (daily steps) alone, ASMI plus age, ASMI plus physical activity (daily steps), and ASMI plus current smoking status. The Poor group was used as the reference group, and group effects were expressed as estimated log_2_-scale effects (log_2_FC), with positive values indicating higher estimated metabolite abundance in the Good group. For univariate analysis, metabolite abundances were log_2_-transformed. Empirical Bayes moderation was used to obtain moderated test statistics, and *P* values were adjusted using the Benjamini–Hochberg procedure to control the false discovery rate (FDR). Differentially abundant metabolites (DAMs) were defined as metabolites meeting both criteria of an FDR-adjusted *P* value < 0.05 and an absolute log_2_FC > 0.25. Ninety-five percent confidence intervals (95% CIs) for the model-estimated log_2_FCs were obtained using the moderated standard errors and moderated degrees of freedom. Group-specific mean ± standard deviation (SD) values were calculated from the log_2_-transformed, unscaled metabolite abundances.

To characterize global metabolic profiles and assess group separation, principal component analysis (PCA) and partial least squares discriminant analysis (PLS-DA) were performed using mixOmics. For PCA, metabolite abundances were log_2_-transformed and Pareto-scaled for exploratory visualization. For PLS-DA, metabolite abundances were log_2_-transformed, and Pareto scaling was performed independently within each training fold, with scaling parameters estimated from the training data only and applied to the corresponding validation or test data. The optimal number of PLS-DA components was selected using nested 5-fold cross-validation, with an inner 5-fold cross-validation used for component selection and an outer 5-fold cross-validation used for performance evaluation. Model discrimination was further assessed using permutation testing with 1,000 permutations, with the complete nested cross-validation procedure repeated for each permutation. Accuracy, balanced accuracy, sensitivity, specificity, and area under the receiver operating characteristic curve (AUC) were calculated. 95% CIs were estimated by bootstrap resampling of the pooled outer-fold predictions. Volcano plots were generated using ggplot2 to visualize metabolite-level effect sizes and statistical significance. DAMs were highlighted using an FDR-adjusted *P* value < 0.05 and an absolute log_2_FC > 0.25. Variable importance in projection (VIP) scores from the PLS-DA model were used to rank metabolites according to their contribution to group discrimination and were interpreted as exploratory measures rather than as criteria for defining DAMs. KEGG pathway enrichment analysis was performed using MetaboAnalystR.

Raw LC–MS data were converted to mzXML format and processed using XCMS (version 3.18.0) in R (version 4.0.0) for feature detection, retention-time correction, and peak alignment. Isotope and adduct annotations were performed using CAMERA (version 1.56.0). Demographic, lifestyle, and body-composition variables were analyzed using GraphPad Prism (version 10.1.2). All statistical analysiswere performed using R (version 4.5.2) and Python (version 3.9.23). Key R packages included limma (version 3.66.0), mixOmics (version 6.34.0), ggplot2 (version 4.0.1), and MetaboAnalystR (version 4.3.0).

## 3 Results

### 3.1 Characteristics of study participants

Baseline characteristics of the total population and the two running-performance groups are presented in **Table 1**. Compared with the Poor group, the Good group had higher daily step counts and ASMI, but lower fat mass and percent body fat (all *P* values < 0.05).

**Table 1:** Baseline characteristics of the study population stratified by 3,000-meter running performance.

Characteristics	Total (n = 141)	3,000-meter running performance		*P* value
		Good (n = 76)	Poor (n = 65)	
Demographics				
Age, median (Q1, Q3), y	33.0 (32.0, 35.0)	33.0 (32.0, 35.0)	33.0 (31.0, 36.0)	0.657
Educational Level, Bachelor’s Degree, n (%)	126 (89.4)	71 (93.4)	55 (84.6)	0.255
Marriage Status, married, n (%)	132 (93.6)	73 (96.1)	59 (90.8)	0.302
Lifestyles				
Current smoking status, yes, n (%)	61 (43.3)	33 (43.4)	28 (43.1)	> 0.999
Physical Activity (daily steps), mean (SD)	10568.7 (5443.3)	12337.9 (5955.5)	8500 (3897.1)	< 0.001
Body Composition				
BMI, median (Q1, Q3), kg/m^2^	24.4 (22.7, 26)	24 (22.5, 25.7)	25.1 (23.3, 26.7)	0.087
Fat Mass, median (Q1, Q3), kg	14.9 (11.4, 18)	12.9 (10.2, 15.3)	17.3 (14.7, 20.1)	< 0.001
Fat Free Mass, mean (SD), kg	58.6 (5.5)	59.2 (5.1)	58 (6.0)	0.215
Percent Body Fat, mean (SD), %	20.2 (5.3)	17.7 (4.4)	23.2 (4.6)	< 0.001
Appendicular Skeletal Muscle Mass Index, median (Q1, Q3), kg/m^2^	11 (10.5, 11.7)	11.2 (10.7, 11.8)	10.9 (10.2, 11.4)	0.004

*Values are presented as mean ± SD, median (Q1, Q3), or n (%), as appropriate. *P* values for continuous variables were calculated using Welch’s t-test (for normally distributed variables: Fat Free Mass, Percent Body Fat, and Physical Activity) or the Wilcoxon rank-sum test (for non-normally distributed variables: Age, BMI, Fat Mass, and ASMI). *P* values for categorical variables were calculated using Fisher’s exact test. Group comparisons were set as Good versus Poor. ASMI, appendicular skeletal muscle mass index; BMI, body mass index; Q1, first quartile; Q3, third quartile; SD, standard deviation.

### 3.2 PCA and PLS-DA model performance and validation

After feature filtering and metabolite annotation, 636 endogenous metabolites were retained for multivariate analysis. PCA showed no clear separation between the Good and Poor groups (**Figure 2A**). PLS-DA showed partial discrimination between the Good and Poor groups. The first and second components explained 2.92% and 2.61% of the variance in the metabolite abundance matrix, respectively. Two components were selected as the optimal number of components for the final PLS-DA model, with the top 10 metabolites ranked by VIP score shown in **Figure 2C** and the top 100 metabolites reported in **Supplementary Table 1**. Permutation testing with 1,000 random label permutations indicated that the observed nested cross-validated classification error was lower than that of the permuted models (observed error rate = 0.4113; mean permuted error rate = 0.5016; empirical *P* = 0.042). In nested five-fold cross-validation, the classification accuracy was 58.9% (95% CI: 51.1–66.7%), with a balanced accuracy of 58.2% (95% CI: 50.0–66.1%), sensitivity of 67.1% (95% CI: 55.8–77.6%), specificity of 49.2% (95% CI: 37.1–62.1%), and an AUC of 0.660 (95% CI: 0.566–0.745).

**Figure 2:**
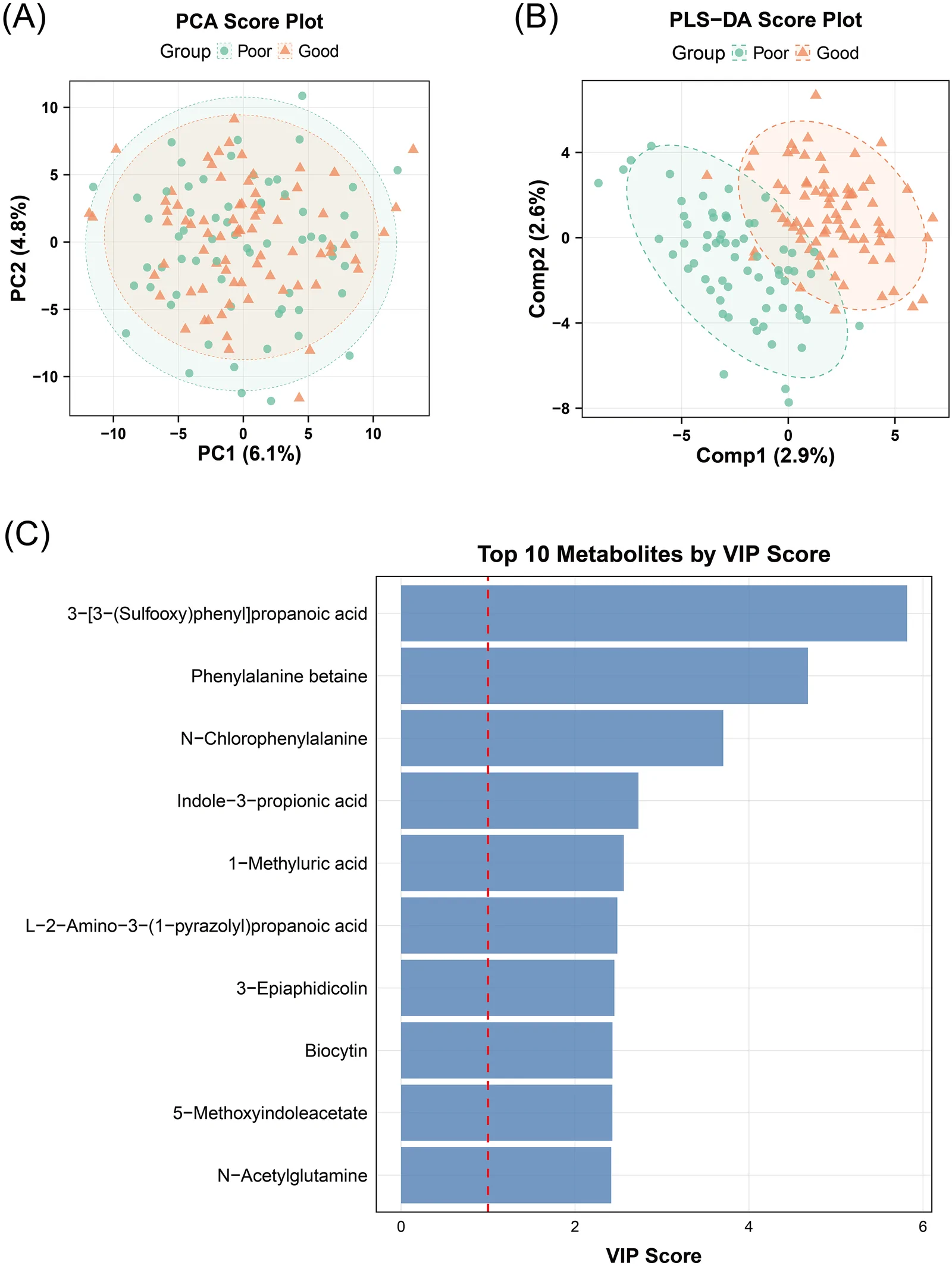
Global serum metabolic profiles and discrimination between the good and poor running-performance groups. **(A)** Principal component analysis (PCA) score plot based on 636 endogenous metabolites. PC1 and PC2 explained 6.1% and 4.8% of the total variance, respectively. **(B)** Partial least squares discriminant analysis (PLS-DA) score plot based on the same 636 endogenous metabolites. The first and second PLS-DA components explained 2.9% and 2.6% of the X-variance, respectively. **(C)** The 10 metabolites with the highest variable importance in projection (VIP) scores in the PLS-DA model. The VIP scores represent the relative contribution of individual metabolites to group discrimination and were used for exploratory ranking.

### 3.3 Identification of 3,000-meter running performance-Associated Metabolites

Covariate-adjusted linear models were fitted separately for all 636 endogenous metabolites, with ASMI included as a continuous covariate. Ten metabolites showed differential abundance between the Good and Poor groups according to the criteria of an FDR-adjusted *P* value < 0.05 and an absolute log_2_FC > 0.25. Among these, four metabolites (i.e., glutamic acid, 3-[3-(sulfooxy)phenyl]propanoic acid, PI(16:0/16:0), and 2-furoylglycine) were higher in the Poor group, whereas six metabolites (i.e., N-chlorophenylalanine, cortisol, L-2-amino-3-(1-pyrazolyl)propanoic acid, 8-hydroxyadenine, piperazine-2,3-dione, and phenylalanine betaine) were higher in the Good group (**Table 2**). The volcano plot of the 10 differential metabolites is shown in **Figure 3A**. Results from the unadjusted model and sensitivity models additionally adjusting for age, physical activity, age plus ASMI, or physical activity plus ASMI are presented in **Supplementary Tables 2**–**8**. The directions of association were consistent across all five models for all 10 metabolites. Four metabolites remained statistically significant across all models, whereas statistical significance varied across adjustment models for the remaining six metabolites.

**Table 2:** Univariate analysis result of 10 significant metabolites, adjusting for ASMI.

No.	Metabolite	*P* value	FDR*	log_2_FC* (95% CI)	Mean ± SD	
					Good	Poor
1	Glutamic acid	<0.001	0.013	-0.262 (-0.380, -0.145)	20.931 ± 0.328	21.146 ± 0.377
2	N-Chlorophenylalanine	<0.001	0.014	0.813 (0.432, 1.195)	18.853 ± 1.073	18.086 ± 1.156
3	3-[3-(Sulfooxy)phenyl]propanoic acid	<0.001	0.031	-1.933 (-2.914, -0.953)	16.006 ± 2.905	17.922 ± 2.783
4	Cortisol	<0.001	0.031	0.321 (0.155, 0.488)	20.037 ± 0.433	19.759 ± 0.545
5	PI(16:0/16:0)	<0.001	0.039	-0.403 (-0.626, -0.179)	21.000 ± 0.735	21.320 ± 0.579
6	L-2-Amino-3-(1-pyrazolyl)propanoic acid	<0.001	0.039	0.385 (0.176, 0.595)	20.006 ± 0.601	19.628 ± 0.613
7	8-Hydroxyadenine	<0.001	0.039	0.381 (0.172, 0.591)	19.825 ± 0.549	19.464 ± 0.673
8	2-Furoylglycine	<0.001	0.039	-0.317 (-0.494, -0.140)	17.890 ± 0.455	18.148 ± 0.599
9	piperazine-2,3-dione	<0.001	0.039	0.259 (0.117, 0.401)	20.107 ± 0.388	19.902 ± 0.460
10	Phenylalanine betaine	<0.001	0.042	1.489 (0.644, 2.335)	22.569 ± 2.034	21.146 ± 2.881

*FDR, Benjamini-Hochberg false discovery rate; log_2_FC, difference in log_2_-transformed metabolite abundance; CI, confidence interval. Poor group was used as the reference. log_2_FC represents the estimated group effect on the log_2_ scale from the corresponding linear model, with positive values indicating higher estimated metabolite abundance in the Good group. Differentially abundant metabolites were defined as FDR-adjusted *P* value < 0.05 and |log_2_FC| > 0.25. Mean ± SD values were calculated from log_2_-transformed, unscaled metabolite abundances.

**Figure 3:**
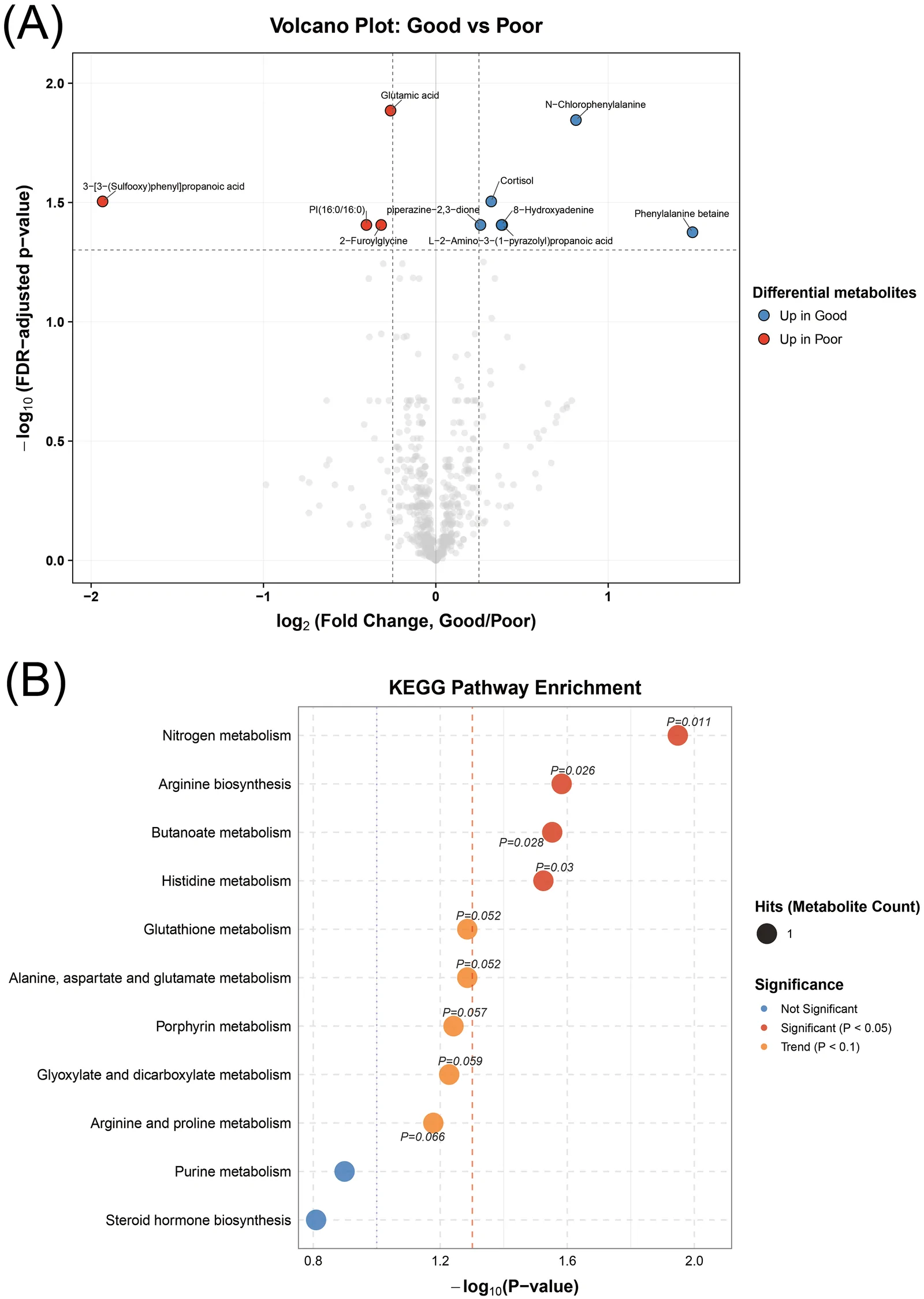
ASMI-adjusted differential metabolites and metabolic pathways associated with running performance. **(A)** Volcano plot showing the associations between serum metabolite abundances and running-performance group after adjustment for ASMI. The x-axis represents the covariate-adjusted log_2_ fold change (log_2_FC; Good versus Poor), with positive values indicating higher abundance in the Good group and negative values indicating higher abundance in the Poor group. The y-axis represents −log_10_ of the Benjamini–Hochberg FDR-adjusted *P* value. Metabolites meeting both criteria of an FDR-adjusted *P* value < 0.05 and |log_2_FC| > 0.25 were considered differentially abundant and are highlighted in color, with blue indicating higher abundance in the Good group and red indicating higher abundance in the Poor group. **(B)** KEGG pathway enrichment analysis based on the 10 differentially abundant metabolites. Point size represents the number of metabolites mapped to each pathway. Pathways with nominal *P* values < 0.05 were considered nominally enriched; none remained significant after FDR correction.

### 3.4 Serum metabolic pathway enrichment analysis

A total of 11 KEGG pathways were identified in the enrichment analysis. Although no pathway remained statistically significant after FDR correction, four pathways showed nominal enrichment (*P* < 0.05) (**Figure 3B**). Nitrogen metabolism showed the strongest nominal enrichment (*P* = 0.011), followed by arginine biosynthesis (*P* = 0.026), butanoate metabolism (*P* = 0.028), and histidine metabolism (*P* = 0.030).

## 4 Discussion

In this cross-sectional study, we characterized serum metabolomic differences associated with running performance in healthy adults residing in cold regions. After accounting for ASMI, significant metabolites were identified between the Good and Poor groups, with corresponding patterns involving amino-acid, phospholipid, stress-, and redox-related metabolism. These findings could raise hypotheses for potential candidate metabolic features associated with differences in running performance, whereas their physiological significance and causal relationships remain to be clarified in future studies.

### 4.1 Amino-acid and nitrogen metabolism in relation to running performance

Among the amino-acid-related metabolites, glutamic acid showed a lower serum concentration in the Good group than in the Poor group after adjustment for ASMI. Glutamate occupies a central position in amino-acid metabolism and is closely connected with the tricarboxylic acid (TCA) cycle through its conversion to α-ketoglutarate, thereby linking amino-acid metabolism with cellular energy metabolism (Muniz-Santos et al., 2024; Kwiatkowski et al., 2025). During exercise, changes in intramuscular glutamate availability can be accompanied by altered uptake from the circulation, particularly in trained individuals (Holeček, 2022). Study evidence suggests that glutamate is recognized as an exercise-responsive metabolite, which has been shown to influence circulating glutamate levels (Xing et al., 2024). In this context, the lower resting serum glutamate concentration observed in the Good group may be consistent with altered glutamate homeostasis or tissue handling. However, this interpretation remains speculative, as circulating glutamate concentrations reflect the balance between production, tissue uptake, interorgan exchange, and nitrogen metabolism.

The nominal enrichment of pathways related to arginine biosynthesis and nitrogen metabolism may nevertheless indicate differences in amino-acid-related metabolic processes between the groups. Previous exercise metabolomics findings showed that arginine biosynthesis, arginine and proline metabolism, and alanine, aspartate and glutamate metabolism were associated with adaptations to aerobic training and gains in maximal power output (Castro et al., 2021). Together, these observations suggest that circulating amino-acid and nitrogen metabolism may be associated with differences in running performance. However, the present cross-sectional design cannot establish whether these differences reflect long-term training adaptation, an inherent metabolic trait, or other unmeasured factors.

### 4.2 Phospholipid remodeling and exercise performance

The lower serum concentration of PI(16:0/16:0), a phosphatidylinositol species, in the Good group suggests differences in phospholipid metabolism and membrane lipid remodeling. Phosphatidylinositol is an important membrane phospholipid and a precursor for phosphoinositide signaling (Dickson and Hille, 2019; Rossignol et al., 2025). Previous intervention studies have reported alterations in circulating phosphatidylinositol species following aerobic exercise, and changes in selected circulating glycerophospholipid species have been associated with improvements in cardiorespiratory fitness (Felder et al., 2017; Zhang et al., 2025). These findings suggest that specific phospholipid species may be responsive to exercise-related metabolic changes, although the direction and magnitude of these changes may differ across molecular species. However, the specific mechanism cannot be determined from circulating metabolite concentrations alone, and the observed reduction should not be interpreted as evidence of either favorable or unfavorable membrane function. Further studies examining tissue lipid composition and phosphatidylinositol-related signaling are needed to determine whether this lipid species has a functional role in endurance performance.

### 4.3 Stress-, redox-, and purine-related metabolic associations

Cortisol, the principal glucocorticoid released through activation of the hypothalamic–pituitary–adrenal axis, plays important roles in energy mobilization, glucose metabolism, and protein turnover (Kraemer et al., 2020; Pérez-Castillo et al., 2023). In the present study, cortisol was more abundant in the Good group. This difference may indicate variation in endocrine or metabolic regulation, although cortisol concentrations are influenced by multiple physiological and environmental factors and cannot be interpreted as a direct marker of better or poorer running performance. 8-hydroxyadenine, an oxidatively modified adenine derivative, was more abundant in the Good group. Oxidative modification of nucleic-acid bases can arise from reactive oxygen species and other oxidizing processes (Hiramoto et al., 1998; Hori et al., 2014), but the biological significance of the observed difference in circulating 8-hydroxyadenine remains uncertain. N-chlorophenylalanine, a chlorinated phenylalanine derivative potentially formed during oxidative chlorination reactions (Freuze et al., 2004), was also more abundant in the Good group. However, the biological significance of this metabolite in relation to exercise performance remains unclear. Nevertheless, these metabolites should not be interpreted as direct markers of oxidative stress or endocrine function, and their physiological significance cannot be established from the present cross-sectional data. Further studies incorporating direct measurements of endocrine function, oxidative stress, and relevant physiological factors are needed to clarify these associations.

### 4.4 Diet- and microbiome-related metabolic signals

Several metabolites potentially related to dietary intake or gut-microbiome-associated metabolism differed between the groups. 2-Furoylglycine was more abundant in the Poor group and has been identified as a candidate biomarker of coffee consumption in nutritional metabolomics studies (Heinzmann et al., 2015). 3-[3-(Sulfooxy)phenyl]propanoic acid, an aryl sulfate metabolite, was also more abundant in the Poor group, although its specific dietary or microbial origins remain uncertain. In contrast, phenylalanine betaine, a betaine-class metabolite, was more abundant in the Good group; betaine compounds have been associated with whole-grain or bran intake and gut microbial metabolism (Koistinen et al., 2019). These findings raise the possibility that diet- and gut-microbiome-associated metabolic processes may contribute to performance-related metabolic phenotypes. However, because dietary intake and gut microbiome composition were not directly assessed in the present study, these interpretations remain exploratory. The observed differences may reflect dietary habits, microbial metabolism, host metabolism, or interactions among these processes, rather than specific dietary or microbiome characteristics of either performance group.

### 4.5 Pathway-level interpretation of metabolic differences

In addition to the nitrogen metabolism and arginine biosynthesis described above, the nominal enrichment of butanoate metabolism and histidine metabolism may be relevant to exercise physiology. Short-chain fatty acids derived from butanoate metabolism have been implicated in the gut–muscle axis and may influence skeletal muscle energy metabolism and inflammatory signaling, while histidine contributes to carnosine synthesis, an important intramuscular buffer during exercise (Perim et al., 2019). These pathway signals suggest that amino-acid, nitrogen, and gut–muscle-related metabolic processes may be associated with differences in running performance. However, pathway-level enrichment does not establish that the corresponding pathways were functionally activated or suppressed in either group. Moreover, given the nominal significance of these findings, they should be interpreted as exploratory signals requiring further validation.

Collectively, the serum metabolomic profile associated with running performance was characterized by differences in amino-acid and nitrogen metabolism, phospholipid metabolism, and stress-, redox-, and diet-related metabolic processes. The observed metabolic differences were not uniformly characterized by higher or lower concentrations in either performance group, highlighting the complexity of the metabolic signatures associated with running performance. These findings should therefore be interpreted as performance-associated metabolic signatures rather than causal determinants.

### 4.6 Limitations

This study has several limitations. First, the cross-sectional design precludes establishing a temporal sequence between running performance and serum metabolic profiles. Second, the cohort was restricted to males to minimize potential sex-related metabolic variability. Therefore, the findings may not be generalizable to females and require validation in more diverse populations. Third, given the limited sample size, we used the model with adjustment for ASMI rather than simultaneously adjusting for a wide range of potential confounders in the main analysis. We also performed sensitivity analysiswith models adjusting for age alone, physical activity (daily steps) alone, ASMI plus age, ASMI plus physical activity (daily steps), and ASMI plus current smoking, and the directions of association for the identified metabolites remained consistent. Nevertheless, residual confounding cannot be completely excluded, particularly from factors that were not assessed in the present study, such as dietary intake and cardiorespiratory fitness (VO_2_max). Future studies with larger sample sizes and more comprehensive assessment of potential confounders are warranted to further clarify these findings.

## 5 Conclusions

In this cross-sectional study, we investigated serum metabolomic profiles in healthy adults residing in cold regions. After accounting for skeletal muscle mass, our analysis identified distinct metabolic patterns associated with differences in running performance between the Good and Poor groups. Our findings suggested that running performance was associated with amino-acid and nitrogen metabolism, phospholipid metabolism, and stress-, redox-, and diet-related metabolic processes. These observations could provide hypotheses of metabolic features associated with running performance in this population, whereas their specific physiological roles and mechanisms require further investigation.

## Data Availability

The raw data supporting the conclusions of this article will be made available by the authors, without undue reservation.
