# Supplementary material for: Serum metabolic signatures associated with running performance in healthy men living in a cold environment: insights into amino acid and energy metabolism
Source: Front Physiol. 2026 Oct 1;17:1920016. doi: 10.3389/fphys.2026.1920016 (PMC13668497; doi:10.3389/fphys.2026.1920016)
Supplement: Supplementary file 1 [file DataSheet1.pdf]

Supplementary Material

Supplementary Table 1. VIP scores of top 100 metabolites from the PLS-DA model.

Supplementary Table 2. Summary of the 10 differential metabolites identified in the primary ASMI-adjusted analysis and their associations with running performance across sensitivity models.

Supplementary Table 3. Unadjusted univariate analysis of differential metabolites.

Supplementary Table 4. Univariate analysis of differential metabolites, adjusting for age.

Supplementary Table 5. Univariate analysis of differential metabolites, adjusting for ASMI and age.

Supplementary Table 6. Univariate analysis of differential metabolites, adjusting for physical activity (daily steps).

Supplementary Table 7. Univariate analysis of differential metabolites, adjusting for ASMI and physical activity (daily steps).

Supplementary Figure 1. PLS-DA model evaluation and metabolite discrimination.

Supplementary Figure 2. Quality control assessment of the metabolomics data.

Supplementary Table 1. VIP scores of top 100 metabolites from the PLS-DA model.

Rank	Metabolite	VIP
1	3-[3-(Sulfooxy)phenyl]propanoic acid	5.81685721
2	Phenylalanine betaine	4.67795871
3	N-Chlorophenylalanine	3.70368430
4	Indole-3-propionic acid	2.72862785
5	1-Methyluric acid	2.56032482
6	L-2-Amino-3-(1-pyrazolyl)propanoic acid	2.48600952
7	3-Epiaphidicolin	2.45226185
8	Biocytin	2.43041276
9	5-Methoxyindoleacetate	2.42941984
10	N-Acetylglutamine	2.41590608
11	11(R)-HETE	2.39137000
12	8-Hydroxyadenine	2.37267965
13	p-Cresol sulfate	2.36386398
14	8,9-DiHETrE	2.36267910
15	Indole	2.30723244
16	Paraxanthine	2.28791329
17	Moxisylyte	2.27563961
18	Caffeine	2.27087284
19	D-Histidine	2.21467518
20	Isoleucyl-Asparagine	2.18677257
21	1-Heptanesulfonic acid	2.15465434
22	N1,N12-Diacetylspermine	2.14801545
23	Cortexolone	2.10893720
24	Norcholic acid	2.09741549
25	Cortisol	2.09417212

26	2-Ethylhexyl acrylate	2.08952683
27	3-(3-Hydroxyphenyl)-2-methylpropionic acid	2.05571905
28	Prostaglandin J2	2.05193596
29	PI(16:0/16:0)	2.02715216
30	3-Oxocholic acid	2.01778427
31	Corticosterone	2.00720584
32	dTDP-D-glucose	1.96548006
33	Vanillin 4-sulfate	1.92232904
34	8-Hydroxyguanine	1.92155500
35	PS(18:0/20:4(8Z,11Z,14Z,17Z))	1.91036730
36	1,7-Dimethyluric acid	1.90619158
37	Leucylalanine	1.89351145
38	Cotinine	1.89039469
39	Indole-3-carboxylic acid	1.88706972
40	Taurocholic acid	1.88055457
41	p-Cresol	1.87949693
42	Isoleucyl-Isoleucine	1.86236995
43	alpha-Bisabolol	1.86194827
44	Glutamic acid	1.86136312
45	3-Hydroxytetradecanoic acid	1.85514687
46	Jasmonic acid	1.85507124
47	2-Furoylglycine	1.83839623
48	16-Hydroxyhexadecanoic acid	1.83421062
49	3-Hydroxydodecanoic acid	1.83248568
50	Phytocassane B	1.80782682
51	PE(16:0/18:1(11Z))	1.80151691
52	3b-Hydroxy-6b-methoxy-7(11)-eremophilen-12,8a-olide	1.76954619
53	Taurochenodesoxycholic acid	1.71892479

54	9Z,12E-Octadecadienoic acid	1.68590818
55	Pipecolic acid	1.68163963
56	PI(16:0/22:2(13Z,16Z))	1.65322266
57	piperazine-2,3-dione	1.64041536
58	Guanine	1.62929207
59	PC(16:0/P-18:1(11Z))	1.61654476
60	N-Methylphenylalanyl-prolyl-arginine	1.61222675
61	Cotinine N-oxide	1.61041867
62	Pantothenol	1.60254049
63	2,3-Epoxyphylloquinone	1.59202121
64	Arginyl-glycyl-aspartic acid	1.58314523
65	12S-HHT	1.57424782
66	Protocatechuic acid 4-O-sulfate	1.56845123
67	Neoporrigenin B	1.56511197
68	Nepetaside	1.56134009
69	12,13-EpOME	1.55841693
70	Docosapentaenoic acid (22n-6)	1.54245432
71	3-Oxotetradecanoic acid	1.54238817
72	LysoPI(18:1(9Z)/0:0)	1.53396466
73	PG(18:0/18:2(9Z,12Z))	1.52951623
74	12-HETE	1.50736909
75	Tridecanoic acid	1.50435121
76	PE(16:0/20:4(5Z,8Z,11Z,14Z))	1.50378993
77	Adipic acid	1.48752865
78	Phenylacetylglutamine	1.47705723
79	Taurodeoxycholic acid	1.47650832
80	delta-CEHC	1.46985564
81	Isocurcumenol	1.46793389

82	Phenylalanylphenylalanine	1.45703090
83	Citpressine II	1.43873225
84	Pyridoxamine	1.41634682
85	Leukotriene B4	1.39643285
86	1,2-Dihexanoyl-sn-glycero-3-phosphoethanolamine	1.39552749
87	4,11,13,15-Tetrahydroridentin B	1.39059510
88	12,13-DHOME	1.38929147
89	Sphingosine 1-phosphate (d16:1-P)	1.38564672
90	L-Arginine	1.38293003
91	PI(18:0/20:3(5Z,8Z,11Z))	1.37634938
92	Indoleacetaldehyde	1.36428528
93	15(16)-EpODE	1.35904083
94	2,6-Dihydroxybenzoic acid	1.35638993
95	1,2-Dioleoyl-sn-glycero-3-phosphoglycerol	1.35428874
96	Chenodeoxycholic acid	1.34381960
97	Threonylphenylalanine	1.34351112
98	N-Acetyl-S-trans,trans-farnesyl-L-cysteine	1.33049237
99	Triethyl Phosphate	1.32668893
100	4-O-Methylgallic acid	1.31077870

Note: VIP, variable importance in projection. VIP scores represent the relative contribution of individual metabolites to the PLS-DA model and were used for exploratory ranking rather than defining differentially abundant metabolites.

Supplementary Table 2. Summary of the 10 differential metabolites identified in the primary ASMI-adjusted analysis and their associations with running performance across sensitivity models.

No.	Metabolite	Adjusting method	*P* value	FDR	$\log_2$FC* (95% CI)
1	Glutamic acid	Unadjusted	<0.001	0.069	-0.213 (-0.332, -0.097)
		Age	<0.001	0.066	-0.217 (-0.335, -0.098)
		ASMI + age	<0.001	0.012	-0.264 (-0.383, -0.146)
		Physical Activity*	<0.001	0.049	-0.233 (-0.359, -0.107)
		ASMI + Physical Activity*	<0.001	0.012	-0.274 (-0.399, -0.149)
		ASMI + Smoking*	<0.001	0.015	-0.258 (-0.376, -0.141)
2	N-Chlorophenylalanine	Unadjusted	<0.001	0.031	0.766 (0.397, 1.135)
		Age	<0.001	0.043	0.740 (0.376, 1.104)
		ASMI + age	<0.001	0.019	0.787 (0.411, 1.163)
		Physical Activity*	<0.001	0.034	0.795 (0.399, 1.190)
		ASMI + Physical Activity*	<0.001	0.012	0.834 (0.429, 1.239)
		ASMI + Smoking*	<0.001	0.015	0.815 (0.432, 1.199)
3	3-[3-(Sulfooxy)phenyl]propanoic acid	Unadjusted	<0.001	0.031	-1.916 (-2.862, -0.971)
		Age	<0.001	0.043	-1.871 (-2.812, -0.929)
		ASMI + age	<0.001	0.037	-1.889 (-2.865, -0.912)
		Physical Activity*	<0.001	0.024	-2.166 (-3.173, -1.159)
		ASMI + Physical Activity*	<0.001	0.012	-2.167 (-3.200, -1.134)
		ASMI + Smoking*	<0.001	0.035	-1.930 (-2.915, -0.945)
4	Cortisol	Unadjusted	0.001	0.084	0.279 (0.116, 0.441)
		Age	0.001	0.099	0.276 (0.112, 0.439)
		ASMI + age	<0.001	0.037	0.319 (0.152, 0.486)
		Physical Activity*	<0.001	0.034	0.338 (0.166, 0.510)
		ASMI + Physical Activity*	<0.001	0.012	0.372 (0.198, 0.547)
		ASMI + Smoking*	<0.001	0.039	0.312 (0.148, 0.476)
5	PI(16:0/16:0)	Unadjusted	0.005	0.212	-0.320 (-0.542, -0.098)
		Age	0.005	0.214	-0.325 (-0.548, -0.102)
		ASMI + age	<0.001	0.037	-0.407 (-0.632, -0.182)
		Physical Activity*	0.004	0.179	-0.355 (-0.592, -0.117)
		ASMI + Physical Activity*	<0.001	0.042	-0.425 (-0.662, -0.188)
		ASMI + Smoking*	<0.001	0.041	-0.402 (-0.627, -0.177)
6	L-2-Amino-3-(1-pyrazolyl)propanoic acid	Unadjusted	<0.001	0.064	0.378 (0.176, 0.580)
		Age	<0.001	0.066	0.365 (0.165, 0.564)
		ASMI + age	<0.001	0.037	0.372 (0.165, 0.579)
		Physical Activity*	<0.001	0.049	0.398 (0.181, 0.614)
		ASMI + Physical Activity*	<0.001	0.042	0.403 (0.181, 0.625)
		ASMI + Smoking*	<0.001	0.041	0.385 (0.175, 0.595)
7	8-Hydroxyadenine	Unadjusted	0.001	0.074	0.361 (0.158, 0.563)
		Age	<0.001	0.080	0.357 (0.154, 0.561)
		ASMI + age	<0.001	0.037	0.378 (0.168, 0.589)
		Physical Activity*	0.002	0.179	0.351 (0.134, 0.568)
		ASMI + Physical Activity*	0.001	0.072	0.369 (0.147, 0.592)

No.	Metabolite	Adjusting method	*P* value	FDR	log_2FC* (95% CI)
		ASMI + Smoking*	0.001	0.041	0.374 (0.165, 0.584)
8	2-Furoylglycine	Unadjusted	0.004	0.209	-0.257 (-0.433, -0.082)
		Age	0.004	0.214	-0.260 (-0.436, -0.084)
		ASMI + age	<0.001	0.037	-0.320 (-0.498, -0.142)
		Physical Activity*	0.003	0.179	-0.285 (-0.472, -0.097)
		ASMI + Physical Activity*	<0.001	0.042	-0.335 (-0.523, -0.147)
		ASMI + Smoking*	<0.001	0.041	-0.314 (-0.492, -0.136)
9	piperazine-2,3-dione	Unadjusted	0.005	0.212	0.205 (0.064, 0.346)
		Age	0.004	0.214	0.207 (0.065, 0.349)
		ASMI + age	<0.001	0.037	0.261 (0.118, 0.404)
		Physical Activity*	0.018	0.349	0.182 (0.031, 0.333)
		ASMI + Physical Activity*	0.003	0.088	0.231 (0.081, 0.380)
		ASMI + Smoking*	0.003	0.041	0.253 (0.112, 0.393)
10	Phenylalanine betaine	Unadjusted	0.001	0.079	1.423 (0.607, 2.239)
		Age	<0.001	0.080	1.430 (0.609, 2.250)
		ASMI + age	<0.001	0.042	1.496 (0.646, 2.345)
		Physical Activity*	0.002	0.179	1.384 (0.509, 2.259)
		ASMI + Physical Activity*	0.002	0.081	1.444 (0.548, 2.341)
		ASMI + Smoking*	0.002	0.041	1.486 (0.637, 2.336)

* Physical Activity, measured by daily steps count; Smoking, current smoking status; FDR, Benjamini-Hochberg false discovery rate; log_2FC, difference in log_2-transformed metabolite abundance; CI, confidence interval. The Poor group was used as the reference; therefore, positive log_2FC values indicate higher abundance in the Good group, whereas negative values indicate higher abundance in the Poor group. Differentially abundant metabolites were defined as FDR-adjusted *P* value < 0.05 and $|log_2FC| > 0.25$.

Supplementary Table 3. Unadjusted univariate analysis of differential metabolites.

No.	Metabolite	*P* value	FDR*	log_2FC* (95% CI)	Mean ± SD	
					Good	Poor
1	3-[3-(Sulfooxy)phenyl]propanoic acid	<0.001	0.031	-1.916 (-2.862, -0.971)	16.006 ± 2.905	17.922 ± 2.783
2	N-Chlorophenylalanine	<0.001	0.031	0.766 (0.397, 1.135)	18.853 ± 1.073	18.086 ± 1.156

* FDR, Benjamini-Hochberg false discovery rate; log_2FC, difference in log_2-transformed metabolite abundance; CI, confidence interval. Poor group was used as the reference. log_2FC represents the difference between the Good and Poor groups. Differentially abundant metabolites were defined as FDR-adjusted *P* value < 0.05 and $|log_2FC| > 0.25$. Mean ± SD values were calculated from log_2-transformed, unscaled metabolite abundances.

Supplementary Table 4. Univariate analysis of differential metabolites, adjusting for age.

No.	Metabolite	*P* value	FDR*	$\log_2$FC* (95% CI)	Mean ± SD	
					Good	Poor
1	3-[3-(Sulfooxy)phenyl]propanoic acid	<0.001	0.043	-1.871 (-2.812, -0.929)	16.006 ± 2.905	17.922 ± 2.783
2	N-Chlorophenylalanine	<0.001	0.043	0.740 (0.376, 1.104)	18.853 ± 1.073	18.086 ± 1.156

* FDR, Benjamini-Hochberg false discovery rate; $\log_2$FC, difference in $\log_2$-transformed metabolite abundance; CI, confidence interval. Poor group was used as the reference. $\log_2$FC represents the difference between the Good and Poor groups after adjustment for age. Differentially abundant metabolites were defined as FDR-adjusted *P* value < 0.05 and $|\log_2\text{FC}| > 0.25$. Mean ± SD values were calculated from $\log_2$-transformed, unscaled metabolite abundances.

Supplementary Table 5. Univariate analysis of differential metabolites, adjusting for ASMI and age.

No.	Metabolite	*P* value	FDR*	log_2FC* (95% CI)	Mean ± SD	
					Good	Poor
1	Glutamic acid	<0.001	0.012	-0.264 (-0.383, -0.146)	20.931 ± 0.328	21.146 ± 0.377
2	N-Chlorophenylalanine	<0.001	0.019	0.787 (0.411, 1.163)	18.853 ± 1.073	18.086 ± 1.156
3	3-[3-(Sulfooxy)phenyl]propanoic acid	<0.001	0.037	-1.889 (-2.865, -0.912)	16.006 ± 2.905	17.922 ± 2.783
4	PI(16:0/16:0)	<0.001	0.037	-0.407 (-0.632, -0.182)	21.000 ± 0.735	21.320 ± 0.579
5	8-Hydroxyadenine	<0.001	0.037	0.378 (0.168, 0.589)	19.825 ± 0.549	19.464 ± 0.673
6	L-2-Amino-3-(1-pyrazolyl)propanoic acid	<0.001	0.037	0.372 (0.165, 0.579)	20.006 ± 0.601	19.628 ± 0.613
7	2-Furoylglycine	<0.001	0.037	-0.320 (-0.498, -0.142)	17.890 ± 0.455	18.148 ± 0.599
8	Cortisol	<0.001	0.037	0.319 (0.152, 0.486)	20.037 ± 0.433	19.759 ± 0.545
9	Phenylalanine betaine	<0.001	0.042	1.496 (0.646, 2.345)	22.569 ± 2.034	21.146 ± 2.881

* FDR, Benjamini-Hochberg false discovery rate; log_2FC, difference in log_2-transformed metabolite abundance; CI, confidence interval. Poor group was used as the reference. log_2FC represents the difference between the Good and Poor groups after adjustment for ASMI and age. Differentially abundant metabolites were defined as FDR-adjusted *P* value < 0.05 and $|log_2FC| > 0.25$. Mean ± SD values were calculated from log_2-transformed, unscaled metabolite abundances.

Supplementary Table 6. Univariate analysis of differential metabolites, adjusting for physical activity (daily steps).

No.	Metabolite	*P* value	FDR*	log_2FC* (95% CI)	Mean ± SD	
					Good	Poor
1	3-[3-(Sulfooxy)phenyl]propanoic acid	<0.001	0.024	-2.166 (-3.173, -1.159)	16.006 ± 2.905	17.922 ± 2.783
2	N-Chlorophenylalanine	<0.001	0.034	0.795 (0.399, 1.190)	18.853 ± 1.073	18.086 ± 1.156
3	Cortisol	<0.001	0.034	0.338 (0.166, 0.510)	20.037 ± 0.433	19.759 ± 0.545
4	L-2-Amino-3-(1-pyrazolyl)propanoic acid	<0.001	0.049	0.398 (0.181, 0.614)	20.006 ± 0.601	19.628 ± 0.613

* FDR, Benjamini-Hochberg false discovery rate; log_2FC, difference in log_2-transformed metabolite abundance; CI, confidence interval. Poor group was used as the reference. log_2FC represents the difference between the Good and Poor groups after adjustment for daily step count. Differentially abundant metabolites were defined as FDR-adjusted *P* value < 0.05 and $|log_2FC| > 0.25$. Mean ± SD values were calculated from log_2-transformed, unscaled metabolite abundances.

Supplementary Table 7. Univariate analysis of differential metabolites, adjusting for ASMI and physical activity (daily steps).

No.	Metabolite	*P* value	FDR*	log_2FC* (95% CI)	Mean ± SD	
					Good	Poor
1	3-[3-(Sulfooxy)phenyl]propanoic acid	<0.001	0.012	-2.167 (-3.200, -1.134)	16.006 ± 2.905	17.922 ± 2.783
2	N-Chlorophenylalanine	<0.001	0.012	0.834 (0.429, 1.239)	18.853 ± 1.073	18.086 ± 1.156
3	Cortisol	<0.001	0.012	0.372 (0.198, 0.547)	20.037 ± 0.433	19.759 ± 0.545
4	Glutamic acid	<0.001	0.012	-0.274 (-0.399, -0.149)	20.931 ± 0.328	21.146 ± 0.377
5	PS(18:0/20:4(8Z,11Z,14Z,17Z))	<0.001	0.042	-0.454 (-0.709, -0.199)	20.177 ± 0.822	20.489 ± 0.574
6	PI(16:0/16:0)	<0.001	0.042	-0.425 (-0.662, -0.188)	21.000 ± 0.735	21.320 ± 0.579
7	L-2-Amino-3-(1-pyrazolyl)propanoic acid	<0.001	0.042	0.403 (0.181, 0.625)	20.006 ± 0.601	19.628 ± 0.613
8	2-Furoylglycine	<0.001	0.042	-0.335 (-0.523, -0.147)	17.890 ± 0.455	18.148 ± 0.599

* FDR, Benjamini-Hochberg false discovery rate; log_2FC, difference in log_2-transformed metabolite abundance; CI, confidence interval. Poor group was used as the reference. log_2FC represents the difference between the Good and Poor groups after adjustment for ASMI and daily step count. Differentially abundant metabolites were defined as FDR-adjusted *P* value < 0.05 and $|log_2FC| >$ 0.25. Mean ± SD values were calculated from log_2-transformed, unscaled metabolite abundances.

Supplementary Table 8. Univariate analysis of differential metabolites, adjusting for ASMI and current smoking status.

No.	Metabolite	*P* value	FDR*	log_2FC* (95% CI)	Mean ± SD	
					Good	Poor
1	N-Chlorophenylalanine	<0.001	0.015	0.815 (0.432, 1.199)	18.853 ± 1.073	18.086 ± 1.156
2	Glutamic acid	<0.001	0.015	-0.258 (-0.376, -0.141)	20.931 ± 0.328	21.146 ± 0.377
3	3-[3-(Sulfooxy)phenyl]propanoic acid	<0.001	0.035	-1.930 (-2.915, -0.945)	16.006 ± 2.905	17.922 ± 2.783
4	Cortisol	<0.001	0.039	0.312 (0.148, 0.476)	20.037 ± 0.433	19.759 ± 0.545
5	Phenylalanine betaine	<0.001	0.041	1.486 (0.637, 2.336)	22.569 ± 2.034	21.146 ± 2.881
6	PI(16:0/16:0)	<0.001	0.041	-0.402 (-0.627, -0.177)	21.000 ± 0.735	21.320 ± 0.579
7	L-2-Amino-3-(1-pyrazolyl)propanoic acid	<0.001	0.041	0.385 (0.175, 0.595)	20.006 ± 0.601	19.628 ± 0.613
8	8-Hydroxyadenine	<0.001	0.041	0.374 (0.165, 0.584)	19.825 ± 0.549	19.464 ± 0.673
9	PI(16:0/22:2(13Z,16Z))	<0.001	0.041	-0.314 (-0.494, -0.135)	23.139 ± 0.447	23.374 ± 0.638
10	2-Furoylglycine	<0.001	0.041	-0.314 (-0.492, -0.136)	17.890 ± 0.455	18.148 ± 0.599
11	piperazine-2,3-dione	<0.001	0.041	0.253 (0.112, 0.393)	20.107 ± 0.388	19.902 ± 0.460
12	Norcholic acid	<0.001	0.047	0.280 (0.117, 0.443)	23.825 ± 0.324	23.598 ± 0.613

* FDR, Benjamini-Hochberg false discovery rate; log_2FC, difference in log_2-transformed metabolite abundance; CI, confidence interval. Poor group was used as the reference. log_2FC represents the difference between the Good and Poor groups after adjustment for ASMI and current smoking status. Differentially abundant metabolites were defined as FDR-adjusted *P* value < 0.05 and $|log_2FC| > 0.25$. Mean ± SD values were calculated from log_2-transformed, unscaled metabolite abundances.

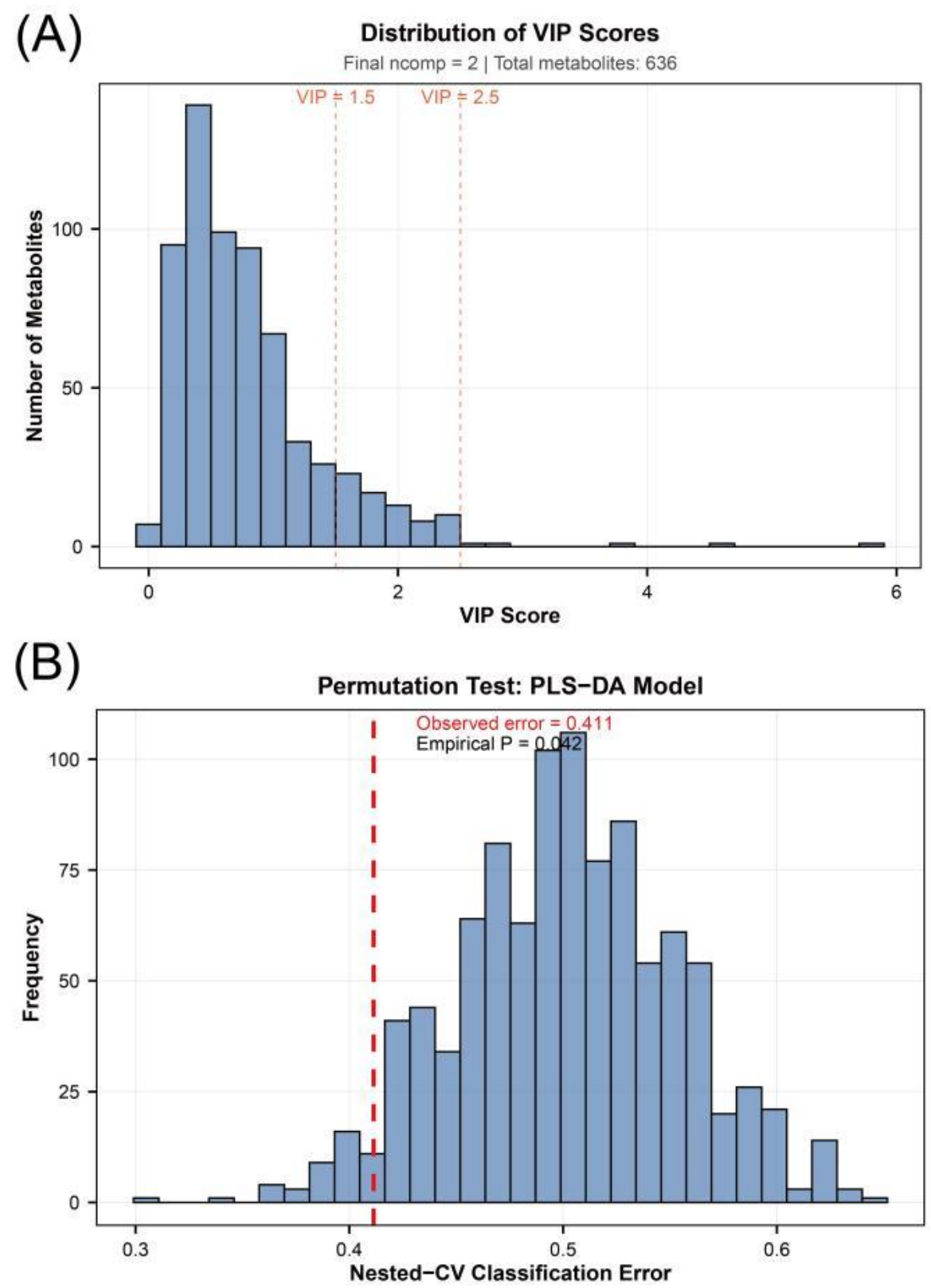

Supplementary Figure 1. PLS-DA model evaluation and metabolite discrimination.

(A) Distribution of variable importance in projection (VIP) scores for the 636 profiled metabolites. The final PLS-DA model was fitted using two components, with the number of components selected through nested cross-validation. (B) Permutation test showing the distribution of classification error rates obtained from 1,000 random permutations of group labels. The red dashed line indicates the observed classification error rate of the original PLS-DA model (41.1%). The observed error rate was lower than the mean error rate obtained under permutation (50.2%), with an empirical *P* value of 0.042.

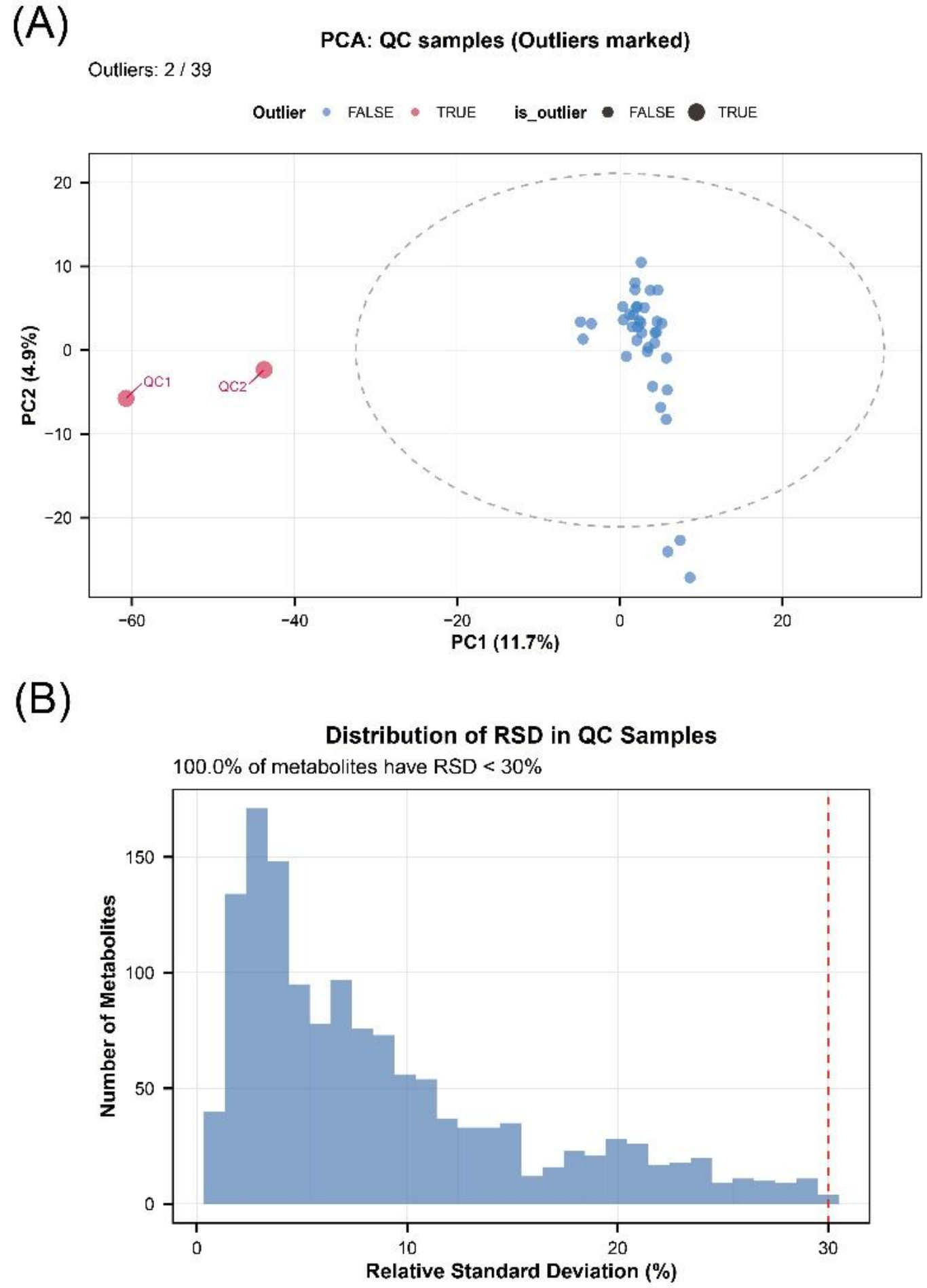

Supplementary Figure 2. Quality control assessment of the metabolomics data.

(A) PCA score plot of quality control (QC) samples. The tight clustering of QC samples indicates good analytical reproducibility. (B) Distribution of relative standard deviation (RSD) values for metabolite intensities across QC samples. The red dashed line indicates the predefined RSD threshold of 30%. The majority of profiled metabolites showed RSD values below 30%, supporting the stability of the analytical platform.
